# Anthracycline dose and liver dysfunction

**DOI:** 10.1038/sj.bjc.6690310

**Published:** 1999-04

**Authors:** R Cannizzaro, D Crivellari, I Robieux, R Sorio, A Buonadonna

**Affiliations:** Department of Gastroenterology, Centro di Riferimento Oncologico IRCCS , Via Pedemontana Occ., Aviano (PN), 33081, Italy; Department of Oncology, Centro di Riferimento Oncologico IRCCS , Via Pedemontana Occ., Aviano (PN), 33081, Italy


*British Journal of Cancer* (1999) **79**(11/12), 1943
© 1999 Cancer Research Campaign
Article no. bjoc.1998.0310

Letter to the Editor

### Anthracycline dose and liver dysfunction

**Sir,**

The issue of anthracycline dose and liver dysfunction is an important one, and we were very interested in the work of Dobbs et al (1998) about oncologist's sensibility for dose modification in patients with liver dysfunction and breast cancer. Usually, current recommendations suggest to use liver function tests, and particularly serum bilirubin, for dose modification. According to our group this issue needs some considerations:

1. Serum bilirubin levels do not reflect a histological damage of liver. If a patient has Gilbert's syndrome, bilirubinaemia levels can increase without significant liver disease (Fevery and Blanckaert, 1991).
2. Conventional so-called liver function tests (AlT, AsT, alkaline phosphatase, GGT, etc.) can reflect liver damage, but they do not correlate with an impairment of liver function. Recent studies observed that quantitative liver function tests can be used for modification of the doses especially because they reflect an underlying liver lesion (Cannizzaro et al, 1996).
3. Treatment with biliary acids (ursodesossicolic acid or others) can be employed to normalize alterated levels of cholestatic indices, such as alkaline phosphatases or GGT, and bilirubinaemia in patients with chronic liver disease and can reduce liver damage (Saksena and Tandon, 1997).

All patients with liver dysfunction are usually excluded from clinical trials using chemotherapy regimens as they have eligibility criteria that require 'normal' liver function, or at best bilirubin values < 1.5 mg dl/l and ALT < 60 IU ml/l. In clinical practice, in the presence of abnormal liver biochemistry, oncologists may be tempted to avoid validated chemotherapy regimens and choose other forms of treatment or to reduce dosage.

'Adjuvant' treatments after surgery are the only ones capable of prolonging disease-free survival and even overall survival, and we know that dose reductions may compromise overall treatment results (Hryniuk et al, 1987). Furthermore, in an attempt to define guidelines for dose modifications it is important in our mind to distinguish different clinical conditions in breast cancer treatment:

1. Liver function tests might be abnormal because of liver invasion by cancer cells (cholestatic indices).
2. The liver dysfunction might be due to underlying chronic liver disease and the patient has a high risk cancer which needs intensive chemotherapy.
3. The liver dysfunction might be due to hepatotoxic changes from chemotherapeutic drugs.

Anthracycline is one the best cytostatic drugs for breast cancer. Instead of reducing the dose intensity, alternative strategies (weekly treatment, for instance) should be considered which permit to reduce tumour burden with small repeated doses of drug. As anthracycline-containing regimens are otherwise increasingly used in adjuvant settings, better dose modification guidelines are urgently needed. On the other hand, we should not forget that breast cancer patients may have a relatively long life-expectancy which could reveal late effects of cytotoxic drugs on liver.

We think there is the urgent need to develop newer systems, including quantitative liver function tests (MEGX test, aminopyrine breath test, etc.) in order to evaluate and monitor liver function during oncological treatments since not only anthracycline, but many anticancer drugs, are excreted by the liver or need it for their activation.

*R Cannizzaro[1], D Crivellari[2], I Robieux[2], R Sorio[2] and A Buonadonna[2]*
*Departments of [1]Gastroenterology and [2]Oncology, Centro di Riferimento Oncologico IRCCS, Via Pedemontana Occ., 33081 Aviano (PN), Italy*

### REFERENCES

Cannizzaro R, Robieux I, Valentini M, Sorio R, Amoroso B, Tumolo S and Campagnutta E (1996) Liver function assessment by MEGX: application to oncology. *Ann NY Acad Sci* **784**: 486–490

Dobbs NA and Twelves CJ (1998) Anthracycline doses in patients with liver dysfunction: do UK oncologists follow current recommendations? *Br J Cancer* **77**: 1145–1148

Fevery J and Blanckaert N (1991) Hyperbilirubinaemia. In *Oxford Textbook of Clinical Hepatology*, McIntyre N, Benhamou JP, Bircher J, Rizzetto M, Rodes J (eds), pp. 985–991. Oxford University Press: Oxford

Hryniuk WM, Bonadonna G and Valagussa P (1987) The effect of dose intensity in adjuvant chemotherapy. In *Adjuvant Therapy of Cancer*, Salmon JE (ed), pp. 13–23. Grusse & Strastton: Orlando

Saksena S and Tandon RK (1997) Ursodeoxycholic acid in the treatment of liver disease. *Postgrad Med J* **73**: 75–80

